# Into the Wild: A Look at *Candida albicans* Outside the Clinical Setting

**DOI:** 10.3390/jof11090622

**Published:** 2025-08-26

**Authors:** Aline Dias Valério, Graciéle Cunha Alves de Menezes, Carlos Augusto Rosa, Susana Johann

**Affiliations:** Departamento de Microbiologia, Instituto de Ciências Biológicas, Universidade Federal de Minas Gerais, Av. Antônio Carlos, 6627, Belo Horizonte P.O. Box 486, 31270-901, MG, Brazilcarlrosa@icb.ufmg.br (C.A.R.)

**Keywords:** *Candida albicans*, natural environment, virulence

## Abstract

*Candida albicans* is a yeast extensively studied for its role in the human microbiota and as a crucial opportunistic pathogen. Despite a vast body of information about this organism, its ecology in natural habitats remains poorly understood. Most studies suggest that *C. albicans* is typically isolated only from endothermic hosts or human-associated substrates. However, several reports have demonstrated the presence of *C. albicans* in environmental samples such as rivers, soils, and plant materials. In this mini-review, we present studies that have isolated *C. albicans* from natural environments and discuss the importance of expanding research efforts to gain a more comprehensive understanding of the ecology of this yeast.

## 1. Introduction

**Figure 1:**
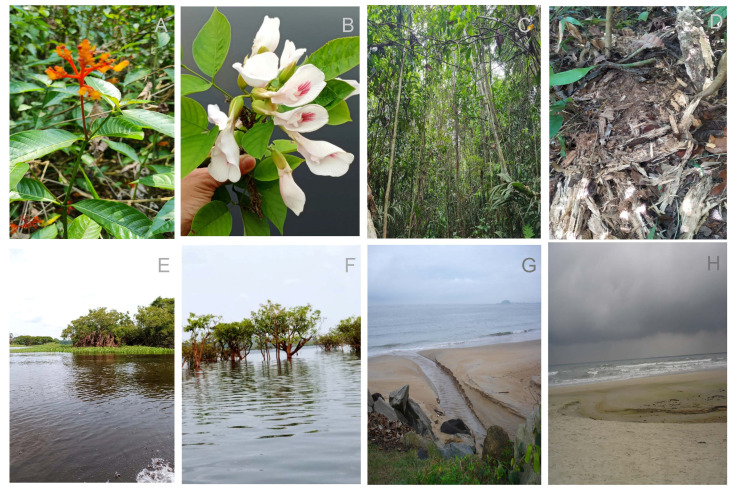
Representation of the ecological habitats where *C. albicans* was found in natural environments. In order from left to right, the pictures include flowers found in the Brazilian Amazon Rainforest (**A**,**B**), trees from the Brazilian Amazon Rainforest (**C**), decomposing wood (**D**), rivers present in the Amazon Rainforest (**E**,**F**), and two beaches from the coastline in Brazil (**G**,**H**). The figures are the property of the authors.

*Candida albicans* is a fungal species that was first described in 1839 by Langenbeck [1]. It belongs to the phylum Ascomycota, subphylum Saccharomycotina, class Pichiomycetes, order *Serinales*, family *Debaryomycetaceae*, and genus *Candida* [2]. Since its first isolation, *C. albicans* has been extensively studied, primarily owing to its role in the human microbiota and as an opportunistic pathogen. As an etiological agent of infections, this microorganism, together with other opportunistic species of the genus *Candida*, accounts for approximately 20% of invasive infections worldwide [3]. However, this number could be significantly higher, given that the diagnosis and identification of fungal infections are generally neglected [3]. Due to the great clinical impact of *C. albicans*, scientific research is focused mainly on its role in the microbiota and as a pathogen. For a long time, this area of study led researchers to believe that this fungus was exclusively present in clinical and endothermic environments [4]. Unlike other *Candida* species, the isolation of *C. albicans* from environmental samples was long considered unlikely. However, some authors have reported that *C. albicans* can occasionally be recovered from environmental substrates, although such occurrences are rare [5,6]. In cases where *C. albicans* was detected in nature, it has often been suggested that these findings may result from contamination during sample collection. However, subsequent research contradicted this, and *C. albicans* was found in several natural environments outside the clinical setting (Figure 1, Table 1). The earliest reports of *C. albicans* isolated from natural environments date back to the mid-20th century, with detections on furze (*Ulex* sp.) flowers and *Myrtus communis* leaves from a hillside grazed by sheep and goats in Portugal [7], as well as on grass in a pasture in New Zealand [8]. Decades later, *C. albicans* was recovered from a broader range of ecological niches, including the flower of an African tulip tree in the Cook Islands and the fruit of *Stenocereus hystrix* (Cactaceae) in Jamaica [6], as well as from oak trees in ancient wood pastures in the United Kingdom [9]. This yeast was later isolated from diverse habitats, including forest soils, decaying wood, rivers, swamps, beaches, and even Antarctic permafrost [4,10,11,12,13,14]. Consequently, there is still a lack of knowledge concerning the phenotypic characteristics expressed by this fungus in natural environments. Furthermore, the behavior of these isolates in the clinical setting and whether the sites of isolation in nature serve as sources of contamination for humans are still uncertain. Given the significance of the subject matter and the scarcity of research on the presence of *C. albicans* in environmental samples, the present study aims to compile available data from the literature and expand our understanding the ecology of this microorganism.

## 2. *Candida albicans* in Soil

Since the 1950s, studies have reported the isolation of *C. albicans* on different types of soil from various parts of the world (Table 1). However, researchers remained uncertain whether these fungi were a result of saprophytic feeding habits or if they originated from human contamination during the collection process. Ajello [15] isolated and identified species of pathogenic fungi, including *C. albicans*, in soil samples collected from regions of North America, Central America, South America and Africa. However, only a single *C. albicans* isolate was obtained from a soil sample collected in Shelby County, Tennessee, USA. Rogers and Beneke [16] identified 21 isolates of *C. albicans* in soil samples collected from various locations across Brazil. Notably, 17 of these isolates were obtained from soils containing cow and hog dung, as well as from chicken house soils on farms near Belo Horizonte (Minas Gerais) and Piracicaba (São Paulo). Two isolates were recovered from cave soil in the Itatiaia Park region (Minas Gerais). One isolate was found in sand near the steps of Santos beach in São Paulo, and another was isolated from flower bed soil in a park in São Paulo. This study was the first to report the isolation of *C. albicans* from soil samples in southern Brazil. However, the authors acknowledged the potential influence of anthropogenic and/or zoonotic factors at the sampling sites, which may have contributed to the presence of *C. albicans* in these environments [16]. Subsequently, another study investigated various samples of Brazilian Amazonian soils as a natural reservoir for pathogenic fungi [12]. *C. albicans* was identified among the species, albeit at low frequency and with random distribution; only a single isolate was found. The authors noted that the material was collected from areas previously studied in their bat research. Interestingly, they reported that the *C. albicans* isolate recovered from the soil was identical to the one previously isolated from internal bat organs. Mok et al. [17] observed that Amazonian soils harbor a rich diversity of yeasts, although most species were found at low frequencies, with random geographical distribution and an apparent lack of species clustering. Interestingly, some of the yeast species recovered from the soil had also been previously isolated from bats in an earlier study by the same research group. The authors noted that while the species compositions of the environmental and animal yeast reservoirs were somewhat similar, they were not identical. Maciel et al. [13] investigated the presence of *C. albicans* and other opportunistic yeasts in sand from different recreational beaches in Brazil and characterized their pathogenic potential. These authors found two isolates of this species during the winter season, one in Rio de Janeiro and another in Paraná. Virulence tests carried out with *C. albicans* isolates revealed their morphogenic capacity, high adhesion rate in oral epithelial cells, biofilm-forming ability, and significant virulence in a murine model. Consequently, the authors suggested a potential risk of contamination by *C. albicans* for beachgoers. The beaches where Maciel et al. [13] conducted their collections were characterized by a high degree of anthropic interference, and it cannot be excluded that human factors may contribute to the presence of *C. albicans* in the collected samples.

*Candida albicans* isolates have also been found at low frequency in soils adjacent to peach trees (*Prunus persica*) in southwestern Slovakia (two isolates) [19], as well as in soils from Iowa and Wisconsin, United States [4]. Based on their findings, Opulente et al. [4] suggest that opportunistic pathogens like *C. albicans* can persist in non-clinical environments, potentially using these habitats on a temporary basis. This implies that the ecology of these yeasts may be more complex than is currently assumed. Using independent cultivation techniques, Sautour et al. [28] developed a nested PCR approach to detect *C. albicans* DNA in soils from different locations in France. Out of the 460 soil samples analyzed in the study, only 7 (1.5%) showed *C. albicans* DNA signatures. In order to understand the parameters that affect yeast survival in French soils, the authors analyzed soil samples and, using Spearman’s correlation test, observed that *C. albicans* was able to survive for up to 30 days in 80% of the soils tested. Sautour [28] demonstrated that the short-term survival of *C. albicans* in soils was associated with certain chemical factors such cation exchange capacity (CEC) and clay content. To analyze this, the authors conducted an experiment using 20 different soil samples and the yeast *Debaryomyces occidentalis*, an environmental yeast employed as a control, which demonstrated prolonged survival in the tested soils. For the *C. albicans* strain, survival depended on soil type: in 11 of the soils, the population even increased after 7 days, particularly in the soil with the most acidic pH (4.9), while the poorest survival occurred in the soil with the highest pH (8.0). A significant effect of lower pH values and exchangeable minerals such as aluminum, manganese, and sodium was observed in enhancing the survival of *C. albicans* in soil after 7 days. Over 30 days, higher cation exchange capacity (CEC) and clay content were identified as the main factors contributing to the long-term survival of *C. albicans* in soils. These findings suggest the existence of potential environmental niches that can support the persistence of this yeast.

## 3. *Candida albicans* in Plants

**Figure 2:**
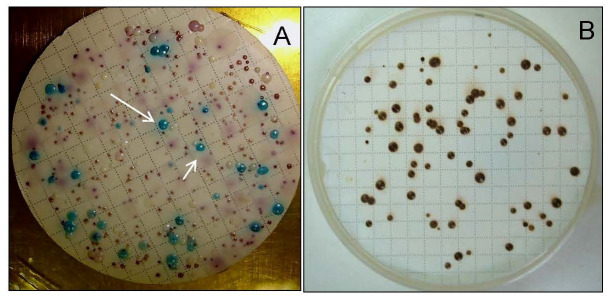
Yeast colonies grown on nitrocellulose membranes using *Candida* CHROMagar-Candida (arrow: *Candida albicans* forming green colonies) (**A**) and MCa media (brown colonies: *C. albicans*) (**B**). The figures are the property of the authors.

Studies suggest evidence that *C. albicans* can exhibit a wide ecological range when isolated from natural sources like plants (Table 1). Van Uden et al. [7] reported the successful isolation of *C. albicans* from floral specimens of *Ulex* sp. (Fabaceae) and *Myrtus communis* (Myrtaceae) in Portugal. In the same study, in vivo tests revealed the high virulence potential of these isolates, causing the death of the rabbits 4 days after infection. Despite these findings, the authors approached, with caution, the saprophytic characteristics exhibited by these yeasts. Although the authors acknowledged the difficulty of definitively confirming plants as a natural habitat, they effectively demonstrated the adaptability of *C. albicans* to survive outside its traditional endothermic hosts. Khan et al. [21] reported the isolation of *C. albicans* from the stem of *Calotropis procera*, a medicinal plant widely used in India. Additionally, the yeast was found associated with decaying wood in the Galápagos Archipelago, Ecuador [22]. However, in both studies, the authors did not delve into the implications of detecting this opportunistic pathogen in these habitats. *C. albicans* isolates were recovered from oak forests in different regions of Europe [9,10]. Robinson et al. [9] isolated *C. albicans* (three strains) from ancient oak trees in northern Europe and suggested that the existence of wild populations of this yeast on northern European trees could potentially explain the previously puzzling maintenance of aquaporin genes associated with freeze tolerance in *C. albicans* [29]. Bensasson et al. [10] compared the genetic characteristics of these oak isolates with yeasts obtained from clinical settings. Their findings revealed that oak isolates predominantly exhibited a diploid nature, similar to clinical isolates. However, *C. albicans* oak isolates showed a higher degree of heterozygosity compared to clinical isolates. Based on these observations, the authors propose that *C. albicans* inhabits the environments of these trees for a prolonged period, showing higher levels of genome-wide heterozygosity. This suggests that they were subjected to different selection or mutation pressures. This study provides insights into the adaptation and long-term evolution of *C. albicans* in distinct ecological niches [10]. Barros et al. [23] explored the diversity of yeasts present in samples isolated from rotting wood in the Amazon forest biomes. *C. albicans* was among the species present in the material collected in the forest. The authors showed, in this study, that the choice of different culture media directly significantly influences the ability to isolate a greater richness of yeast species. This insight leads us to believe that with more comprehensive monitoring, a greater quantity and variety of yeasts can be discovered in the environment. Lopes et al. [12] also found the presence of *C. albicans* associated with rotting wood samples obtained from Brazilian ecosystems, from the areas of Atlantic rainforest (three isolates), Cerrado (one isolate), and Amazonian Forest (one isolate). The number of *C. albicans* isolates found by the authors was not high in each region; however, the authors were using selective media for isolation of cellobiose-fermenting yeast species. Perhaps if more specific media for *C. albicans* isolation were used, such as MCa medium or CHROMagar-*Candida *(Figure 2), more isolates of this species could be found in these biomes [8,30].

## 4. *Candida albicans* in Freshwater and Sea

**Figure 3:**
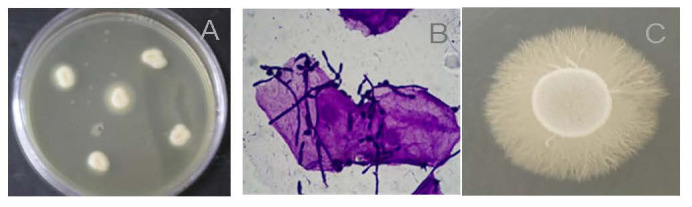
*Candida albicans* egg culture medium exhibited the presence of a phospholipase halo (arrow) (**A**). *C. albicans* adhered to oral epithelial cells in the presence of blastoconidia, hyphae, and pseudohyphae (**B**). Colonies of *C. albicans* grown in Spider culture medium exhibited filamentation ability (**C**). The figures are the property of the authors.

Isolation of *C. albicans* in water bodies has been considered by many researchers as indicative of a contamination site by human wastes (Table 1). In studies where the diversity of microorganisms in seawater from Brazilian beaches was verified, *C. albicans* was frequently isolated at low densities [8,31]. However, in the evaluation of virulence factors expressed by these environmental isolates, Maciel et al. [13] discovered the ability of morphogenesis and biofilm formation through both in vitro and in vivo experiments (Figure 3). The authors highlighted that these results demonstrated the potential of yeasts isolated from environmental samples to maintain their virulence and induce disease.

Striving to identify the sources of microbiological pollution that may harm the health of bathers, Prigitano et al. [18] investigated the fungal populations in both the sands and water of the beaches of two largest Italian lakes and in sands of the Mediterranean coast in Southern Italy. The survey revealed the existence of opportunistic pathogenic fungi in both locations, with *C. albicans* being exclusively detected in the coastal regions at low density. As part of an effort to collect data on fungi in beach sand and water, both in coastal bathing areas and inland waters, Brandão et al. [20] isolated several fungi known as opportunistic human pathogens, such as species of the genus *Aspergillus, Cryptococcus* and *Candida*. Notably, among the findings of this study, *C. albicans* was also isolated from samples of sand and seawater from beaches in the Mediterranean, Black Sea and Northwest Europe regions.

*C. albicans* isolates were also obtained from water samples from different rivers used for human consumption across parts of the world [24,25,26]. Kulesza et al. [25], in a 20-year retrospective study, found that the presence of this opportunistic pathogen is more prominent in areas in Poland where the river flows close to cities, indicating anthropic influence. Monopathi et al. [26] observed the genetic similarity between efflux pump genes (associated with resistance traits) between environmental and clinical isolates, indicating that the environmental isolates already possess resistance to antifungals such as azoles. In both studies, the authors emphasize that these findings represent crucial epidemiological factors with potential implications for clinical environments. The presence of this opportunistic pathogen in these rivers can pose significant risks to people and animals that come into contact with and/or consume this water, especially concerning immunosuppressed individuals. *C. albicans* isolates were recovered from groundwater in residential wells located in regions of the state of Mato Grosso do Sul, Brazil [27]. This study observed that the *C. albicans* isolates produced hemolysin, a recognized key virulence factor, along with phospholipase, facilitating the host cell invasion process (Figure 3). Cupozak-Pinheiro et al. [27] highlight the importance of verifying the presence of potentially pathogenic/virulent yeasts in residential wells, which could lead to opportunistic infections in individuals consuming this water. In addition to running water environments, swampy environments can act as reservoirs for *C. albicans*. Water samples were collected from various depths in a swampy river in South Africa, and the presence of *C. albicans* was detected using quantitative real-time PCR (qRT-PCR) [14]. The river under investigation was contaminated by sewage, indicating clear anthropogenic influence on the findings. The study suggested that oxygen-limited, reducing zones of wetlands act as a niche for *C. albicans* outside its human host and a potential external reservoir for this human commensal. The authors emphasize the epidemiological importance of identifying these reservoirs of opportunistic pathogens outside the hospital environment for disease management.

## 5. Clinical Importance of the Isolation of *Candida albicans* in Natural Environments

Studies involving environmental yeasts, including *C. albicans*, are invaluable for advancing knowledge in ecology, taxonomy, and biotechnological applications. However, specifically regarding the study of environmental isolates of *C. albicans*, it is essential to consider the potential implications for clinical settings. Studies have demonstrated that investigating environmental isolates can significantly enhance our comprehension of the evolutionary events that underlie fungal virulence (Figure 3) [32,33,34]. Notably, studies conducted by Steenbergen et al. [32], Casadevall et al. [33], and Lemos Tavares et al. [34] have unveiled the crucial role of interaction between soil-dwelling amoebae and various fungal species, including *C. albicans*, in shaping the exhibited virulence factors of these fungi. These studies suggest that some virulence factors present in these microorganisms may have emerged through selective pressure during interaction with amoebas in natural settings, highlighting the intricate evolutionary processes at play. For instance, filamentation, which is believed to have evolved as a defense mechanism against amoebae predation, has now been recognized as a crucial factor enabling fungal evasion of the human immune system and tissue invasion. In addition to being a possible tool that would assist in the study of the evolution of virulence factors in fungi, research focused on environmental isolates of opportunistic pathogens can be useful in assessing the behavior of these microorganisms in the face of climate change. Studies on the subject show that climate change can influence host–pathogen interactions, disease spread, antimicrobial resistance, and virulence of these microorganisms [35,36,37,38,39]. Leach et al. [38] found a relationship between the transcription factors responsible for inducing gene expression of genes linked to heat shock proteins and the induction of the transcriptional program associated with the virulence factors of *C. albicans*.

This study reinforces the idea that exposure to stress conditions, such as rising temperature, can influence the expression of genes associated with virulence factors in this fungus. Resistance to antifungal treatment may be another factor that can be assessed in environmental isolates. Research has shown that opportunistic species of the genus *Candida* (*Loderomyces/Candida albicans* clade) obtained in nature may be resistant to treatment with antifungal agents, even without previous contact with these drugs [8,40,41,42,43,44,45]. Understanding the mechanisms involved in this characteristic can help researchers anticipate the emergence of multidrug-resistant isolates and the advancement of novel antifungal drugs.

## 6. Conclusions

There exists a knowledge gap concerning the ecological aspects of opportunistic pathogens, particularly *C. albicans*. The pursuit of knowledge in the clinical domain tends to overlook the significance of comprehending the characteristics of these microorganisms present in non-endothermic host environments. The impact of isolating *C. albicans* from natural habitats on the epidemiology of opportunistic infections remains unclear. Limited data exist regarding the expression of virulence factors by these microorganisms in the wild. It is unknown whether selective pressures in these environments influence traits that serve as secondary sources of contact, leading to contamination and human infections. Essentially, our understanding of *C. albicans* in the non-clinical setting is insufficient. Consequently, it is imperative to invest in research within this field to provide data on its unknown ecology, which may be linked to its clinical pathogenicity. Expanding our knowledge of the natural reservoirs of these yeasts is crucial to gaining a better understanding of the pathophysiology of exogenous infections.

## Figures and Tables

**Table 1:** Isolates of *Candida albicans* from different habitats.

Number of Isolates	Substract/Habitat	Site	City/Country	Reference
1	Soil	Jefferson	Iowa, USA	Opulente et al. [4]
1	Soil	Northeastern	Wisconsin, USA	Opulente et al. [4]
1	Soil	Northeastern	Wisconsin, USA	Opulente et al. [4]
1	Plant Matter Soil	Mesa Canyon San Antonio	Texas, USA	Opulente et al. [4]
1	Plant Matter Soil/Duff	Mesa Cyn San Antonio, TX	Texas, USA	Opulente et al. [4]
1	Soil	Shelby County	Tenesesse, USA	Ajello et al. [15]
8	Soils containing cow and hog dung	Farms near Belo Horizonte	Minas Gerais, Brazil	Rogers and Beneke [16]
8	Chicken house soils	Farms near Belo Horizonte	Minas Gerais, Brazil	Rogers and Beneke [16]
2	Cave soil	Itatiaia Park area	Minas Gerais, Brasil	Rogers and Beneke [16]
1	Chicken yard soil	Piracicaba and Vicinity	Piracicaba, São Paulo	Rogers and Beneke [16]
1	Sand near steps	Santos beach area	Santos, Brasil	Rogers and Beneke [16]
1	Park flower bed soil	São Paulo	São Paulo, Brazil	Rogers and Beneke [16]
1	Amazonian soils	Brazilian Amazon Basin	Brazilian Amazon Basin, Brazil	Mok et al. [17]
3	Sand	Beach	Paraná and Rio de Janeiro, Brazil	Maciel et al. [13]
28.3 CFU/g	Sands	Sicilian Costal beach	Italy	Prigitano et al. [18]
2	Soil near peach tree	Southwest of Slovakia	Slovakia	Vadkertiová et al. [19]
1.7 CFU/g	Sands	Irlanda	Irlanda	Brandao et al. [20]
5.0 CFU/g	Sands	Black sea	Romania	Brandao et al. [20]
27.1 CFU/g	Sands	Mediterranean	Serbia	Brandao et al. [20]
3.3 CFU/g	Sands	Mediterranean	Turkey	Brandao et al. [20]
1	Bark tree	Wyalusing State Park	Wisconsin, USA	Opulente et al. [4]
1	Plant Matter—Fruit; Ericaceae Berry	West Sand Island	Oregon, USA	Opulente et al. [4]
1	Plant Matter—Fruit; Ericaceae Berry	Hungarian Falls	Michigan, USA	Opulente et al. [4]
1	Plant Matter—Fruit; Ericaceae Berry	Hungarian Falls	Michigan, USA	Opulente et al. [4]
1	Flower of African tulip tree (*Spathodea campanulate*, Bignoniaceae)	Rarontohga	Cook Islands	Lachance et al. [6]
1	Fruit of *Stenocereus hystrix* (Cactaceae)	-	Jamaica	Lachance et al. [6]
1	Leaves of *Myrtus communis*	Near the top of a hill (300 m. high) near Vermoil	Estremadura, Portugual	Van Uden et al. [7]
2	Flowers of furze (*Ulex* sp.)	Near the top of a hill (300 m. high) near Vermoil	Estremadura, Portugual	Van Uden et al. [7]
3	Oak	New Forest, North Europe	UK	Robinson et al. [9]
1	Steam of *Calotropis procera* (Ait.) R. Br.	Karachi University campus	Pakistan	Khan et al. [21]
2	Rotting wood samples	Galápagos Archipelago	Ecuador	Guama’n-Burneo et al. [22]
1	Rotting wood	Brazilian Amazonian rainforests	Brazil	Barros et al. [23]
3	Rotting wood	Atlantic Rainforest	Brazil	Lopes et al. [12]
1	Rotting wood	Cerrado	Brazil	Lopes et al. [12]
1	Rotting wood	Amazonian rainforests	Brazil	Lopes et al. [12]
2	Seawater	Recreational beaches	Paraná, Brazil	Maciel et al. [13]
5 CFU/ml	Water	Sicilian Costal beach	Italy	Prigitano et al. [18]
3.8 CFU/ml	Water	Northwest Europe	Irland	Brandao et al. [20]
3.8 CFU/mL	Water	Mediterranean	Serbia	Brandao et al. [20]
25 and 10% *	Water	Blue Nile River	Sudan	Bakhiet et al. [24]
**	Water	Łyna River	Olsztyn, Poland	Kulesza et al. [25]
37	Water	North West Province Rivers	South Africa	Monopathi et al. [26]
1	Water	Groundwater for human consumption from wells	Mato Grosso do Sul, Brazil	Cupozak-Pinheiro et al. [27]

* In this study, the authors reported that *Candida albicans* was found in 25% and 10% of the samples using two different culture media. ** In this study, the authors did not specify how many *Candida albicans* isolates were found.

## References

[1] Gow N., Yadav B. (2017). Microbe Profile: Candida albicans: A shape-changing, opportunistic pathogenic fungus of humans: This article is part of the Microbe Profiles collection. Microbiol..

[2] Groenewald M., Hittinger C.T., Bensch K., Opulente D., Shen X.-X., Li Y., Liu C., LaBella A., Zhou X., Limtong S. (2023). A genome-informed higher rank classification of the biotechnologically important fungal subphylum *Saccharomycotina*. Stud. Mycol..

[3] Lamoth F., Lockhart S.R., Berkow E.L. (2018). Changes in the epidemiological landscape of invasive candidiasis. J. Antimicrob. Chemother..

[4] Opulente D.A., Langdon Q.K., Buh K.V., Haase M.A.B., Sylvester K., Moriarty R.V., Jarzyna M., Considine S.L., Schneider R.M., Hittinger C.T. (2019). Pathogenic budding yeasts isolated outside of clinical settings. FEMS Yeast Res..

[5] Barnett J.A. (2008). A history of research on yeasts 12: Medical yeasts part 1, *Candida albicans*. Yeast.

[6] Lachance M.-A., Boekhout T., Scorzetti G., Fell J.W., Kurtzman C.P., Kutrzman C., Fell J.W., Boekhout T. (1923). 2011 Chapter 90 *Candida* Berkhout. The Yeasts.

[7] Van Uden N., De Matos Faia M., Assis-Lopes L. (1956). Isolation of *Candida albicans* from vegetable sources. J. Gen. Microbiol..

[8] Di Menna M. (1958). *Candida albicans* from Grass Leaves. Nature.

[9] Robinson H.A., Pinharanda A., Bensasson D. (2016). Summer temperature can predict the distribution of wild yeast populations. Ecol. Evol..

[10] Bensasson D., Dicks J., Ludwig J.M. (2019). Diverse lineages of *Candida albicans* live on old oaks. Genetics.

[11] Kochkina G., Ivanushkina N., Ozerskaya S. (2012). Ancient fungi in Antarctic permafrost environments. FEMS Microbiol. Ecol..

[12] Lopes M.R., Lara C.A., Moura M.E.F., Uetanabaro A.P.T., Morais P.B., Vital M.J., Rosa C.A. (2018). Characterization of the diversity and physiology of cellobiose-fermenting yeasts isolated from rotting wood in Brazilian ecosystems. Fungal Biol..

[13] Maciel N.O., Johann S., Brandão L.R., Kucharíková S., Morais C.G., Oliveira A.P., Freitas G.J., Borelli B.M., Pellizzari F.M., A Santos D. (2019). Occurrence, antifungal susceptibility, and virulence factors of opportunistic yeasts isolated from Brazilian beaches. Mem. Inst. Oswaldo Cruz..

[14] Stone W., Jones B.L., Wilsenach J., Botha A. (2012). External ecological niche for Candida albicans within reducing, oxygen-limited zones of wetlands. Appl. Environ. Microbiol..

[15] Ajello L. (1956). Soil as natural reservoir for human pathogenic fungi. Science.

[16] Rogers A.L., Beneke E.S. (1964). Human pathogenic fungi recovered from Brasilian soil. Mycopathol. Mycol. Appl..

[17] Mok W.Y., Luizão R.C., Do Socorro Barreto Da Silva M., Teixeira M.F., Muniz E.G. (1984). Ecology of pathogenic yeasts in Amazonian soil. Appl. Environ. Microbiol..

[18] Prigitano A., Trovato L., Esposto M.C., Brandão J., Cogliati M., Gatta G.D., Grancini A., Migliorisi G., Oliveri S., Romanò L. (2023). Fungal diversity in lake and sea beaches of Italy: Relevance to human health. Sci. Total Environ..

[19] Vadkertiová R., Dudášová H., Stratilová E., Balaščáková M. (2019). Diversity of yeasts in the soil adjacent to fruit trees of the Rosaceae family. Yeast.

[20] Brandão J., Gangneux J.P., Arikan-Akdagli S., Esposto M.C., Brandão J., Cogliati M., Gatta G.D., Grancini A., Migliorisi G., Oliveri S. (2021). Mycosands: Fungal diversity and abundance in beach sand and recreational waters—Relevance to human health. Sci. Total Environ..

[21] Khan R., Shahzad S., Choudhary M.I., Khan S.A., Ahmad A. (2007). Biodiversity of the endophytic fungi isolated from *Calotropis procera* (AIT.) R. BR. Pak. J. Bot..

[22] Guamán-Burneo M.C., Dussán K.J., Cadete R.M., Cheab M.A., Portero P., Carvajal-Barriga E.J., da Silva S.S., Rosa C.A. (2015). Xylitol production by yeasts isolated from rotting wood in the Galápagos Islands, Ecuador, and description of *Cyberlindnera galapagoensis* f.a., sp. nov. Antonie Van Leeuwenhoek.

[23] Barros K.O., Alvarenga F.B.M., Magni G., Souza G.F.L., Abegg M.A., Palladino F., da Silva S.S., Rodrigues R.C.L.B., Sato T.K., Hittinger C.T. (2023). The Brazilian Amazonian rainforest harbors a high diversity of yeasts associated with rotting wood, including many candidates for new yeast species. Yeast.

[24] Bakhiet S., Ahmed W., Mohammed W. (2016). Significance of fungal species isolated from blue nile river and tuti island on drinking water quality. J. Appl. Life Sci. Int..

[25] Kulesza K., Biedunkiewicz A., Nowacka K., Glinka P. (2018). Potentially pathogenic fungi of the *Candida* genus isolated from the Łyna River—A 20-year study. Ann. Parasitol..

[26] Monapathi M.E., Bezuidenhout C.C., Rhode O.H.J. (2018). Efflux pumps genes of clinical origin are related to those from fluconazole-resistant *Candida albicans* isolates from environmental water. Water Sci. Technol..

[27] Cupozak-Pinheiro W.J., Araújo De Almeida-Apolonio A., Sasaki M.H., Maran N.H. (2022). *Candida* species contamination in drinking groundwater from residence wells in three municipalities of midwestern Brazil and the potential human health risks. Microb. Pathog..

[28] Sautour M., Lemaître J., Ranjard L., Truntzer C., Basmaciyan L., Depret G., Hartmann A., Dalle F. (2021). Detection and survival of *Candida albicans* in soils. Environ. DNA.

[29] Tanghe A., Carbrey J.M., Agre P., Thevelein J.M., Van Dijck P. (2005). Aquaporin expression and freeze tolerance in *Candida albicans*. Appl. Environ. Microbiol..

[30] Buck J.D., Bubucis P.M. (1978). Membrane filter procedure for enumeration of *Candida albicans* in natural waters. Appl. Environ. Microbiol..

[31] Pinto K.C., Hachich E.M., Sato M.I.Z., Di Bari M., Coelho M.C.L.S., Matté M.H., Lamparelli C.C., Razzolini M.T.P. (2012). Microbiological quality assessment of sand and water from three selected beaches of South Coast, São Paulo State, Brazil. Water Sci. Technol..

[32] Steenbergen J.N., Nosanchuk J.D., Malliaris S.D., Casadevall A. (2004). Interaction of *Blastomyces dermatitidis*, *Sporothrix schenckii*, and *Histoplasma capsulatum* with *Acanthamoeba castellanii*. Infect. Immun..

[33] Casadevall A., Fu M., Guimaraes A., Albuquerque P. (2019). The ‘amoeboid predator-fungal animal virulence’ hypothesis. J. Fungi.

[34] Lemos Tavares P., Carvalho Ribeiro A., Kercher Berte F. (2020). The interaction between *Sporothrix schenckii* sensu stricto and *Sporothrix brasiliensis* with *Acanthamoeba castellanii*. Mycoses.

[35] Altizer S., Ostfeld R.S., Johnson P.T.J., Kutz S., Harvell C.D. (2013). Climate change and infectious diseases: From evidence to a predictive framework. Science.

[36] Raffel T.R., Romansic J.M., Halstead N.T., McMahon T.A., Venesky M.D., Rohr J.R. (2013). Disease and thermal acclimation in a more variable and unpredictable climate. Nat. Clim. Change.

[37] Maynard J., Van Hooidonk R., Eakin C.M., Puotinen M., Garren M., Williams G., Heron S.F., Lamb J., Weil E., Willis B. (2015). Projections of climate conditions that increase coral disease susceptibility and pathogen abundance and virulence. Nat. Clim. Change.

[38] Leach M.D., Farrer R.A., Tan K., Miao Z., Walker L.A., Cuomo C.A., Wheeler R.T., Brown A.J.P., Wong K.H., Cowen L.E. (2016). Hsf1 and Hsp90 orchestrate temperature-dependent global transcriptional remodelling and chromatin architecture in *Candida albicans*. Nat. Commun..

[39] Cavicchioli R., Ripple W.J., Timmis K.N., Azam F., Bakken L.R., Baylis M., Behrenfeld M.J., Boetius A., Boyd P.W., Classen A.T. (2019). Scientists’ warning to humanity: Microorganisms and climate change. Nat. Rev. Microbiol..

[40] Medeiros A.O., Kohler L.M., Hamdan J.S., Missagia B.S., Barbosa F.A., Rosa C.A. (2008). Diversity and antifungal susceptibility of yeasts from tropical freshwater environments in Southeastern Brazil. Water Res..

[41] Brandão L.R., Medeiros A.O., Duarte M.C., Barbosa A.C., Rosa C.A. (2010). Diversity and antifungal susceptibility of yeasts isolated by multiple-tube fermentation from three freshwater lakes in Brazil. J. Wather Health.

[42] Brilhante R.S.N., Castelo Branco D.S.C.M., Duarte G.P.S., Paiva M.A., Teixeira C.E., Zeferino J.P., Monteiro A.J., Cordeiro R.A., Sidrim J.J., Rocha M.F. (2012). Yeast microbiota of raptors: A possible tool for environmental monitoring: Use of *Candida* spp. for environmental monitoring. Environ. Microb. Rep..

[43] Castelo-Branco D.S.C.M., Brilhante R.S.N., Paiva M.A.N., Teixeira C.E.C., Caetano E.P., Ribeiro J.F., Cordeiro R.A., Sidrim J.J.C., Monteiro A.J., Rocha M.F.G. (2013). Azole-resistant *Candida albicans* from a wild Brazilian porcupine (*Coendou prehensilis*): A sign of an environmental imbalance?. Med. Mycol..

[44] Brilhante R.S.N., Paiva M.A.N., Sampaio C.M.S., Castelo-Branco D.S.C.M., Alencar L.P., Bandeira T.J.P.G., Cordeiro R.A., Neto W.d.A.P., Moreira J.L.B., Sidrim J.J.C. (2015). Surveillance of azole resistance among *Candida* spp. As a strategy for the indirect monitoring of freshwater environments. Water Air Soil. Pollut..

[45] Rocha M.F.G., Alencar L.P., Paiva M.A.N., Melo L.M., Bandeira S.P., Ponte Y.B., Sales J.A., Guedes G.M.M., Castelo-Branco D.S.C.M., Bandeira T.J. (2016). Cross-resistance to fluconazole induced by exposure to the agricultural azole tetraconazole: An environmental resistance school?. Mycoses.

